# Possible metabolic interaction between docetaxel and ifosfamide

**DOI:** 10.1054/bjoc.1999.0949

**Published:** 2000-01-18

**Authors:** Y Ando

**Affiliations:** First Department of Internal Medicine, Nagoya University School of Medicine, 65 Tsurumai-cho, Showa-ku, Nagoya 466, Japan


*British Journal of Cancer* (2000) **82**(2), 497
© 2000 Cancer Research Campaign
Article no. bjoc.1999.0949

Letters to the Editor

### Possible metabolic interaction between docetaxel and ifosfamide

**Sir**

I read with interest the report by Pronk and colleagues on a dose escalation study of the combination chemotherapy of docetaxel and ifosfamide (Pronk et al, 1998). They evaluated an alternating sequence of ifosfamide 24-h infusion and docetaxel. Docetaxel preceded 1 h before ifosfamide administration, and in the reverse ifosfamide was followed by docetaxel with a 24-h interval. They described that the maximum tolerated dose (MTD) was higher when docetaxel was administered before ifosfamide than vice versa and that this schedule was recommended for phase II studies.

I would be of the opinion that the sequence with higher MTD is not always optimal. In vitro studies using human liver microsomes showed that cytochrome P450 3A4 should be involved in both metabolic pathways by detoxification of docetaxel and activation of ifosfamide (Chang et al, 1993; Marre et al, 1996). This implies that the metabolic activation of ifosfamide might be competitively inhibited by the preceded docetaxel with the short interval. In other words, the sequence with the higher MTD might result in a lower anti-tumour activity than the reverse at the same doses of the drugs. Other combination regimens, for example docetaxel plus cisplatin or paclitaxel plus cyclophosphamide seem unlikely to cause such interaction because of their different metabolic pathways. The combination of docetaxel and ifosfamide, however, substantially differs from them.

A primary endpoint of phase I study is generally to find the optimal dose of tested drugs, not to evaluate antitumour activity. In this case, I consider that further phase II studies of both schedules should be essential to determine the optimal sequence. Otherwise, it would be inevitable to show the reason of their decision based on pharmacokinetic data including active metabolites of ifosfamide.

*Y Ando*
*First Department of Internal Medicine,*
*Nagoya University School of Medicine,*
*65 Tsurumai-cho, Showa-ku,*
*Nagoya 466, Japan*

### REFERENCES

Chang TKH, Weber GF, Crespi CL and Waxman DJ (1993) Differential activation of cyclophosphamide and ifosfamide by cytochromes P-450 2B and 3A in human liver microsomes. *Cancer Res* **53**: 5629–5637

Marre F, Sanderink GJ, de Sousa G, Gaillard C, Martinet M and Rahmani R (1996) Hepatic biotransformation of docetaxel (taxotere) in vitro: involvement of the CYP3A subfamily in humans. *Cancer Res* **56**: 1296–1302

Pronk LC, Schrijvers D, Schellens JHM, de Bruijn EA, Planting ASTh, Locci-Tonelli D, Groult V, Verweij J and van Oosterom AT (1998) Phase I study on docetaxel and ifosfamide in patients with advanced solid tumours. *Br J Cancer* **77**: 153–158

